# Examining the effects of pre-competition rapid weight loss on hydration status and competition performance in elite judo athletes

**DOI:** 10.1038/s41598-023-41872-1

**Published:** 2023-09-07

**Authors:** Dawid Bialowas, Radoslaw Laskowski, Emerson Franchini, Sylwester Kujach

**Affiliations:** 1 Department of Physiology, Gdansk University of Physical Education and Sport, Gorskiego 1, 80-336 Gdansk, Poland; 2 Martial Arts and Combat Sports Research Group, Sport Department, School of Physical Education and Sport, University of Sao Paulo, Sao Paulo, Brazil; 3 Department of Neurophysiology, Neuropsychology and Neuroinformatics, Medical University of Gdansk, Tuwima 15, 80-210 Gdansk, Poland

**Keywords:** Physiology, Nutrition

## Abstract

The prevalence of rapid weight loss (RWL) among martial arts athletes including judo is very high. Many applied RWL strategies could be dangerous to health and even lead to death. Therefore, the International Judo Federation (IJF) introduced changes in the weigh-in rules, changing the official weigh-in for the day before the competition. Thus, the purpose of this study was to examine the impact of the new IJF rules on hydration status and weight loss strategies among professional judo athletes. Seventeen elite judo athletes participated in the study. Body mass and hydration status, were analyzed before the competition. Moreover, competition result and practice of RWL survey were collected. All subjects reached their weight category limits for the competition. RWL resulted in body mass changes (*p* < 0.001, η_p_^2^ = 0.79) and dehydration among participants (urine osmolality > 700 [mOsmol_*_kg]^−1^ and urine specific gravity > 1.020 [g_*_cm^3^]^−1^). However, urine osmolality (*p* > 0.05, η_p_^2^ = 0.18), as well as urine specific gravity (*p* > 0.05, η_p_^2^ = 0.16), at subsequent time points of measurement revealed no statistical differences. The prevalence of RWL was 100%, and only 17.6% of the athletes declared that they would compete in a different weight category if the competition would be conducted on the same day of the weigh-in. All judo athletes applied RWL procedures using traditional methods to achieve the required body mass (i.e., increased exercise, reduced fluid, and food intake). Dehydration state was not associated with competitive performance (*p* > 0.05).

## Introduction

Judo is a grappling Olympic combat sport characterized by dynamic, high-intensity intermittent actions, and that requires complex skills development and tactical excellence for success^1–5^. In judo, athletes are classified according to their body mass in order to assure that the opponents have similar body size, strength and agility^6,7^. However, in an attempt to compete against lighter and potentially smaller and weaker adversaries, judo athletes use various methods to manipulate their body mass to qualify in a lower weight category to obtain a competitive advantage^8^. Rapid weight loss (RWL) involves swiftly decreasing a significant amount of body mass within a short duration, often pursued to align with specific weight category criteria for competitive events^9–11^. RWL usually begins in the week leading up to the competition, with the highest amount of body mass being reduced in the last 3 days^10–12^. This reduction is achieved by using aggressive nutritional and physiological methods that influence fluid-electrolyte turnover and whole-body water balance^11–14^. Studies investigating the prevalence of RWL among judo athletes have revealed extremely high values, reaching 89%^11^ up to 100% when judo athletes participating in the world ranking competitions were considered^15^. The most common methods used by athletes are sauna bathing and wearing plastic clothing, fluid and caloric restriction, and the intake of diuretics and laxatives^11^. Shortly after athletes intentionally reduce their body mass to meet specific weight category requirements, they initiate rapid weight regain (RWG) strategies following the official weigh-in, resulting in a swift and considerable increase in body mass^9,16^. Interestingly it has been shown recently that catabolic hormonal reactions that occurred during the calorie restriction were maintained after the RWG intervention. RWL significantly decreases testosterone and thyroid-stimulating hormone concentration among Muay Thai fighters. Additionally, no restoration after the RWG process was observed. Moreover, the above mentioned hormonal modulation may affect behavioral changes such as self-confidence, fighting motivation, competition anxiety, or mental toughness^17^. These strategies can also potentially lead not only to diminished physical performance but also causes health risks, especially when considered that young judo athletes also engage in such practices^6^. In more severe cases, deaths have been reported in athletes using RWL procedures, likely due to heat stress, dehydration and hyperthermia. Thus, for safety reasons changes in weight control programs of combat sports were suggest several times^18,19^. The National Collegiate Athletic Association (NCAA) initiated new rules, to prevent resulting from RWL tragedies, by checking athletes hydration status at the time of weight certification, and authors reported decreased RWL procedures and rapid weight gain in wrestlers^20^.

The International Judo Federation (IJF) also introduced changes in the weigh-in rules, changing the official weigh-in for the day before competition, and introducing a random weigh-in in the competition day, allowing judo athletes to be up to 5% above the upper-limit of their weight category^21^. Therefore, considering these new rules judo athletes likely began to select optimal strategies to reduce their body mass up to the official weigh-in and then recover as much as possible up to the 5% limit. However, little is known regarding the impact of these new rules on the RWL, RWG and hydration status in judo athletes in the days approaching the competition, at the weigh-in and at the competition. So far, only one study was found investigating the hydration status of judo athletes according to the new weigh-in rules^22^. These authors measured the hydration status of eight judo athletes one week before, at the official weigh-in and 24 h post-competition. They reported that athletes reduced 6.8% of their body mass from one week up to the official weigh-in, and increased 4.2% of their body mass between the official weigh-in up to 24 h post-competition. They also reported a significant difference in urine specific gravity between the official weigh-in (1.030 ± 0.001) and 24 h post-competition (1.017 ± 0.007), indicating they were still dehydrated 24 h post-competition^22^. However, it is important to emphasize that this study was conducted with only 8 judo athletes and did not include a measurement in the competition day; therefore, studies with a higher sample size, and measurements in the competition day are needed. Thus, the purpose of the present study was to examine the effect of new IJF rules on RWL, RWG, and hydration status of the judo athletes during a national competition, as well as their performance. We hypothesized that even an extended time to achieve the body mass required by the rules would not ensure the safe application of RWL/RWG strategies. Moreover, the dehydration that often accompanies rapid weight loss due to limited fluid intake and exposure to heat would affect competition performance.

## Results

**Figure 1:**
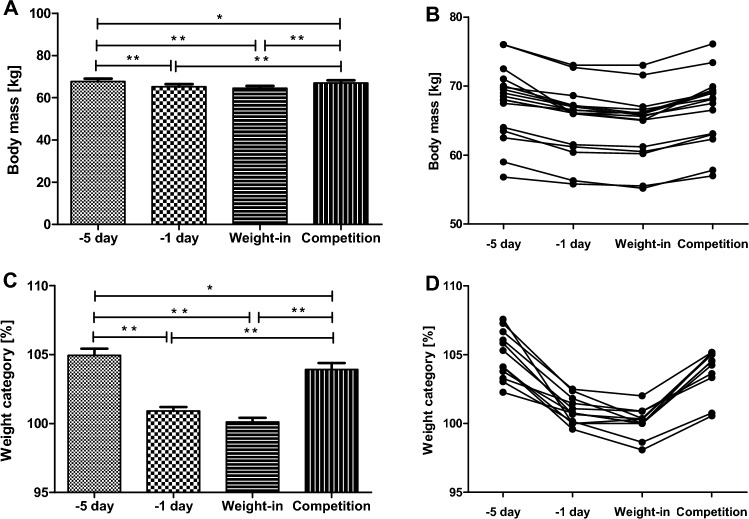
Body mass changes [kg] (**A**,**B**) and body mass changes relative to the upper limit of the athlete’s weight category [%] (**C**,**D**) within the five days prior to the competition. Values displayed on panels (**A**,**C**) are means, and panel (**B**,**D**) represents individual data for each subject. Error bars indicate ± SD. *p < 0.05; **p < 0.01.

The analysis revealed statistical differences in body mass between the following time points (F(3,48) = 62.59; *p* < 0.001 η_p_^2^ = 0.79 [large effect]). A post-hoc Bonferroni test analysis: 5 day vs. − 1 day: *p* < 0.001; − 5 day vs. Weigh-in: *p* < 0.001; − 5 day vs. Competition: *p* = 0.04; − 1 day vs. Competition: *p* < 0.001; Weigh-in vs. Competition: *p* < 0.001 (Fig. 1 A,B). Moreover, there was a significant difference between body mass changes relative to the upper limit of the athlete’s weight category following time points (F(3,48) = 67.23; *p* < 0.001; η_p_^2^ = 0.80 [large effect]). A significant progressive body mass loss from the fifth day before the competition to the day of the competition was found: − 5 day vs. − 1 day: *p* < 0.001; − 5 day vs. Weigh-in: *p* < 0.001; − 5 day vs. Competition: *p* = 0.03; − 1 day vs. Competition: *p* < 0.001; Weigh-in vs. Competition: *p* < 0.001 (Fig. 1C,D).

**Figure 2:**
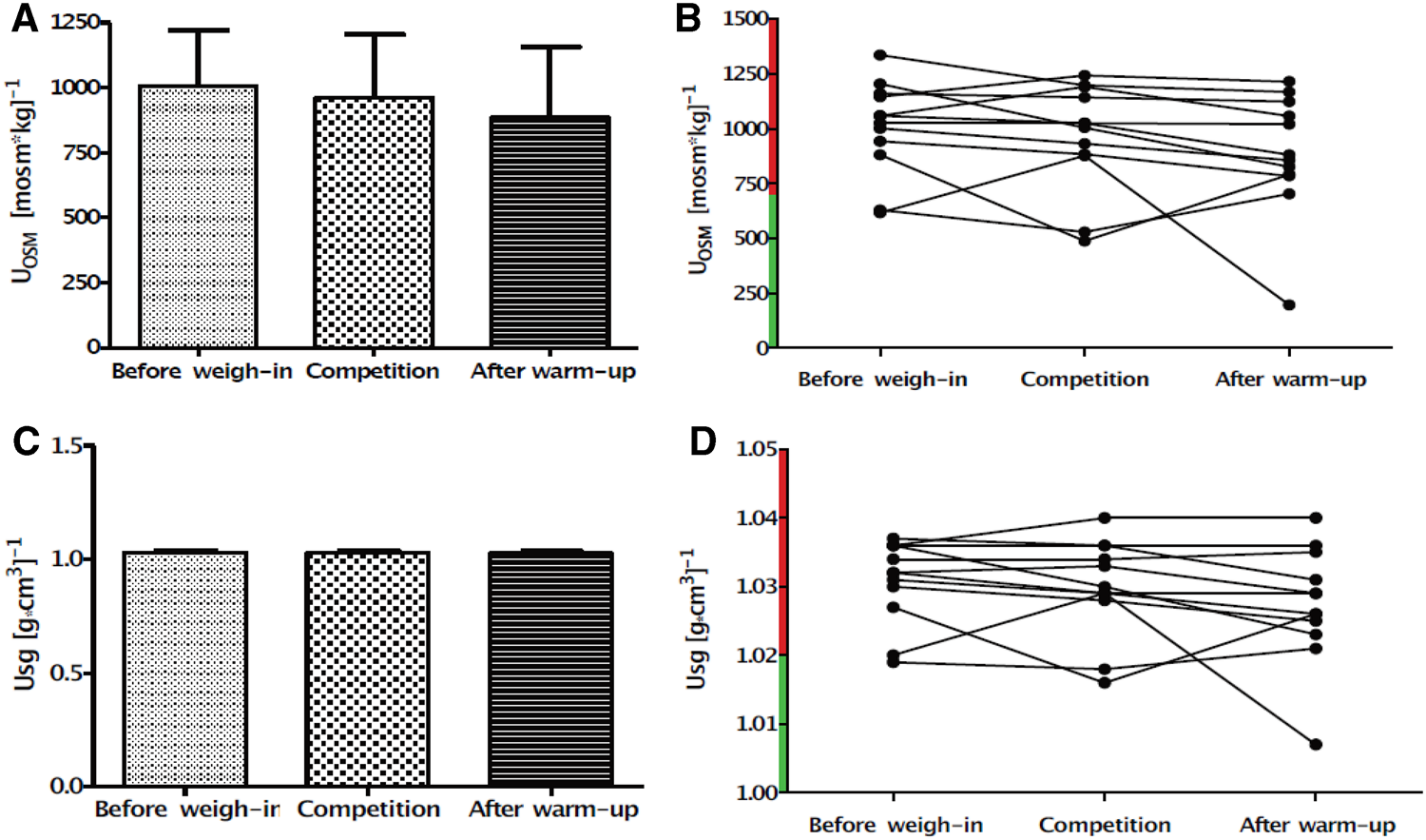
Urine osmolality (U_OSM_) (**A**,**B**) and Urine specific gravity (U_sg_) (**C**,**D**) changes before weigh-in, competition day, and after warm-up. Values displayed on panels (**A**,**C**) are means, and panel (**B**,**D**) represents individual data for each subject. A red highlighted axis on panels (**B**,**D**) indicates dehydration. Error bars indicate ± SD.

For hydration status determination the urine osmolality (U_OSM_) and urinary specific gravity (U_SG_) have been analyzed. The analysis revealed no statistical differences in U_OSM_ as well as U_SG_ along time points of measurement (*p* = 0.10; F_(2,22)_ = 2.48; η_p_^2^ = 0.18., *p* = 0.14; F_(2,22)_ = 2.14; η_p_^2^ = 0.16 respectively) (Fig. 2).

**Figure 3:**
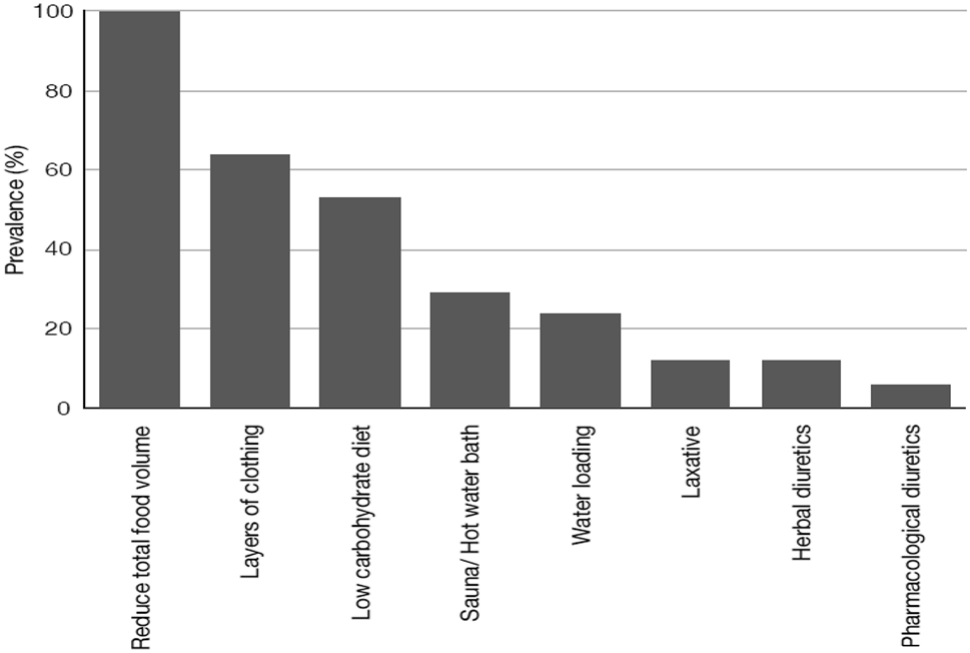
Rapid weight loss (RWL) methods among participants (%).

The prevalence of RWL was 100%, and only 17.6% of the athletes declared they would compete in a different weight category if the competition would be conducted in the same day of the weigh-in. The most common methods of RWL were increased exercise and decreased food/fluid intake. Seven of athletes (41%) declared that they had worked with registered dietitian before the event. Nevertheless, 70.5% of all participants consumed liquids, during the competition day, only if they were thirsty (Fig. 3).

**Figure 4:**
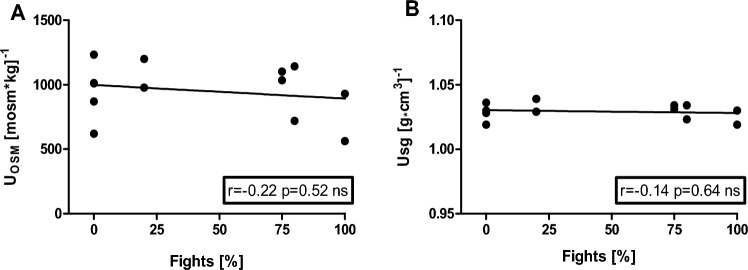
Association between competitive performance expressed in the percentage of possible to win fights and the dehydration state expressed in the average values of all measurement time points for Urine osmolality (U_OSM_) (**A**) and Urine specific gravity (U_sg_) (**B**).

Additionally, we performed Spearman correlation analysis to examine the association between competitive performance and dehydration state in U_OSM_ (r = − 0.22, *p* = 0.52), and U_sg_ (r = − 0.40, *p* = 0.64) (Fig. 4).

## Discussion

The main findings of the present study indicated that all athletes who participated in this investigation practiced RWL, and that the most used methods were increased exercise and decreased fluid and food intake. All athletes achieved the limit of their weight category. Body mass, expressed in absolute (kg) or relative to the weight category upper limit (%), was higher 5-days before competition compared to 1-day and 10-h before competition, and higher in the competition day compared with 1-day and 10-h before the weigh-in. However, UOSM and USG did not vary during the period of measurements, and were significantly correlated (r = 0.68). Conversely, percentage of matches won did not correlate significantly with any body mass, UOSM or USG variables.

The fact that all athletes in the present study used RWL strategies is similar to a recent finding with judo athletes taking part in the competitions of the judo world ranking system (i.e., 100%)^15^, and higher than what was previously reported when the weigh-in was conducted in the same day of the competition (i.e., 89%)^11^. The high prevalence of athletes using RWL strategies may be due to the weigh-in rule change, which now allow the athletes to recover during 15–17 h instead of the usual 3–4 h allowed before. Thus, it is likely that more judo athletes felt that they could recover within this time period, especially because a 5% body mass tolerance is provided now. Additionally, judo athletes may have observed that a high percentage of the athletes is using RWL, and not using it would result in a disadvantage in competition. This perception that using RWL may result in competitive advantage^23^ and the cascade effect was already observed and criticized by some authors^24^. However, the magnitude of RWL conducted by the judo athletes in the present study (~ 5%) was similar to observed in other studies^15^, but slightly less than the only study available analyzing body mass changes in international level judo athletes along the RWL and competition^22^. This amount of reduction is probably related to two aspects: (a) weight categories in judo present nearly 10% increments in body mass. Thus, athletes in the middle of two weight categories limits are more prone to reduce their body mass instead of trying to increase it; (b) per rule, athletes can be 5% above the weight category upper-limit in the day of competition. Therefore, those reducing nearly 5% will in fact compete in their “walking weight”. However, it is important to consider that, in the present study, body mass measurements were conducted from 5 days to the competition up to the competition, and some athletes may have started to reduce their body mass before this period. Conversely, only 17.6% of the judo athletes declared they would participate in a tournament in a heavier weight category if the official weigh-in was conducted in the day of competition, suggesting that for most of the athletes the decision regarding using RWL procedures is not affected by the moment of the weigh-in.

The methods used for athletes in our sample were similar to previously reported for judo athletes from different countries^25–27^. As some investigations^28^ observed that older athletes and coaches are among the most influential people regarding the practice of RWL by judo athletes, it seems that the use of these methods is passed through generations of athletes. Therefore, educational programs should aim at increasing the athletes, coaches and parents awareness about nutritional strategies of healthier ways to properly manage body mass reduction and its regain after weigh-in. Indeed, 41% of the athletes reported to be oriented by a registered dietitian (41%), which is higher compared to other studies^14,29^.

However, the advice of experts usually ended up on body mass reduction without specific intervention regarding rehydration after the weigh-in and for the competition day, what was reflected in the survey. As consequence of the lack of education concerning these aspects, judo athletes started competition in a dehydrated condition and were still dehydrated at the end of event. Most of the athletes in our study reported that they were drinking fluids only when being thirst, but it seems that the physical and psychological stress during the competition day made the athletes to ignore the signs of dehydration such as thirst. Moreover, intense exercise in heavy clothing, like warming up wearing judogi (judo suit) and other sportswear, can cause further progressive reduction of the plasma volume^30^.

The fact that UOSM and USG did not change during the period of measurements indicate that the judo athletes investigated are either constantly mildly dehydrated or started their body mass reduction more than 5 days before the competition. Independently of the reason for the absence of change on these variables, it is important to emphasize that athletes were dehydrated along all the time-points analyzed, i.e., even during the competition day, when they were allowed to recover up to 5% of their body mass (even though they recovered ~ 4%). The amount of body mass recovered by the athletes in the present study was similar to that reported in a recent study analyzing international-level judo athletes post-competition (4.2%)^22^. For that study, however, judo athletes presented a lower USG 24-h post-competition compared with the weigh-in measurement^22^. Thus, the additional 24-h given in the study by Ceylan et al. may have contributed to this higher re-hydration^22^.

No correlation was found between any of body mass, UOSM, and USG variables and percentage of matches won. This differs from the study from Reale et al., that found that body mass regain was higher in medal winners than non-medal winners for male and female judo athletes, and males only^31^. However, these authors did not observe any difference for body mass regain in the first round matches, even though winners had a higher body mass regain than losers when all matches were considered. The fact that the judo athletes in the present study recovered more than double of their body mass (~ 4%) compared with the athletes analyzed by Reale et al., (1.5% for the males sample), suggest that most of them were basically with the same body mass during the competition^31^. Thus, a low variation (95% CI 3.1; 4.7%) observed with the athletes of our study was too small to affect competitive performance. However, the small sample in the present study is a limitation to determine the influence of RWL and RWG on judo competitive performance. Considering the high prevalence of RWL observed in our study and the methods used during the RWL process, it is important that athletes, coaches and competition managers be educated regarding more effective and scientifically-based approaches^17,32^, and that weight management control programs, such as those suggested by Artioli et al. be implemented^18^.

This study is not without limitations. Firstly, a small sample size was applied. Secondly, the study focused solely on male participants. Thirdly, the absence of a control group (non-RWL) restricts comprehensive analysis. However, both enlarging the sample size and incorporating female athletes present notable challenges. Our study was conducted before and during major national sports competitions, during which athletes are subjected to a multitude of factors that might impact their performance. As a result, they tend to be hesitant about participating in such experiments. Furthermore, due to variations in the menstrual cycle timing (often disrupted by extreme training loads), which can influence hormonal changes regulating fluid balance and body mass, studying female athletes becomes even more complex^33^. Nevertheless, future experiments should consider increasing the sample size and including female athletes as well as comparing the RWL with the non-RWL group.

## Conclusions

In conclusion, our findings indicated that all judo athletes applied RWL procedures, using traditional methods to achieve the body mass required (i.e., increased exercise, reduced fluid and food intake), resulting in a dehydration status from 5 days before the competition up to the competition day. However, RWL and RWG were not associated with competitive performance. Moreover, athletes and coaches should be educated regarding more effective and scientifically-based approaches to body mass management and control programs. Also, to minimize the risk of excessive dehydration and potential health deterioration, it is advisable to engage the expertise of an experienced sports nutritionist.

## Methods

### Participants

Seventeen healthy male judo athletes (age: 18.8 ± 0.5 years; height: 172 ± 5 cm; judo grade: 1st kyu and 1st dan) participated in the study. The judo athletes were multiple participants in national competitions both in the youth and senior categories. Moreover, four subjects also competed in international championship competitions level. The study was announced by research staff (former Judo athletes) through social media, and information provided to the coaches. The rules of the national judo federation required a medical check-up for being admitted to the competition. No athlete had a major medical disorder or was taking medication at the time of measurement. Participants competed in four weight categories: 55 kg (n = 2), 60 kg (n = 3), 66 kg (n = 10) and 73 kg (n = 2). All participant declared to use RWL procedures to this event. All the subjects provided written informed consent prior to the study procedures. All the procedures were approved by the Bioethical Committee of the Regional Medical Society (KB-10/16). The study was conducted in accordance with the Declaration of Helsinki The present study ensured anonymity and confidentiality by replacing the athletes personal identification (Supplementary Tables).

### Study design

**Figure 5:**
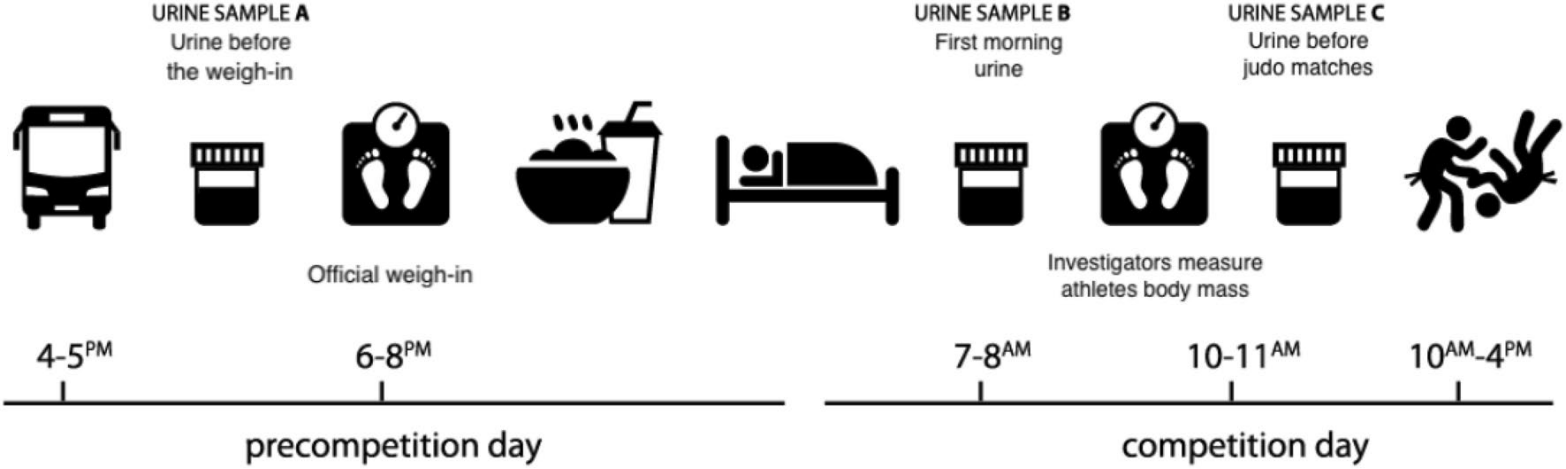
Overview of the measurement protocol. Urine sample A—collected before the official weigh-in; Urine sample B—collected at the morning of the competition day; Urine sample C—collected after end of warming-up athletes, before judo matches; Body mass control—collected at the same time as sample C collection; Body mass was measured together with official weigh-in control and at the time of warming up.

Body mass and body composition were estimated using a multi-frequency impedance plethysmograph body composition analyzer (Tanita BC—545N, Japan). Body mass was measured together with official weigh-in control and after of warming up time, and also expressed relative to the upper-limit of the athlete’s weight category. This measurement was conducted in all athletes in each time-point. To meet objectives, body mass, hydration status, competition result, and practice of RWL survey were also collected. Hydration status was determined by measuring changes in urine osmolality (U_OSM_) and urinary gravity (U_SG_). Among the available methods for assessing hydration levels, blood osmolality stands as the "gold standard"^34,35^. However, measuring blood osmolality requiers an invasive approach and skilled medical personnel, making it challenging for scientists and coaches. In light of these constraints, urine analysis has been proposed as an alternative indication for hydration status due to its noninvasive nature^36^. Moreover, more recent findings indicate that urine closely reflects blood responses during progressive dehydration induced by exercise^37^. Urine can be assessed based on attributes like density, osmolality, or its components, as well as color^38^. U_OSM_ emerges as a non-invasive surrogate for blood osmolality and is recognized as the most credible measure of hydration status through urine^35,39^. Additionally U_OSM_ and U_SG_ has been used, as generally accepted markers of hydration status in the field^6,40^. To differentiate euhydration from dehydration, American College of Sports Medicine (ACSM) and National Athletic Trainers’ Association position (NATA) cut off standard was used, which is U_OSM_ > 700 (mOsmol/kgH2O) and U_SG_ > 1.020 (g/dL)^41^. All participants were instructed about the proper execution of the procedures and collected midstream urine samples into a polypropylene container. Samples were collected before official weigh-in (− 0 to 2 h) (sample A), first void (urination) of the morning, following and overnight fast (sample B), and after warming-up-before the judo matches (sample C). Hospital laboratory completed all remaining urine tests. These two measurements were conducted in 12 athletes. Five athletes were unable to urinate likely due to the severe dehydration and this measurement is lacking for them. All athletes achieved their weight category body mass limit, and none was eliminated in the random weigh-in in the competition day (Fig. 5).

### Statistical analyses

Data are presented as mean and standard deviation. Body mass, UOSM and USG along the rapid weight loss process and recovery were compared through a one-way analysis of variance with repeated measurements, after the confirmation of the compound symmetry via the Mauchly test. Bonferroni test was used as post-hoc when a difference was found in the analysis of variance. Significance level was set at 5%. Correlations between variables were evaluated using the Spearman correlation coefficient. Effect sizes were assessed via partial eta squared (ηp2), using 0.1, a small effect; 0.3, a medium effect; and 0.5, a large effect respectively^42^.

### Supplementary Information

Supplementary Tables.

## Data Availability

Data may be available by email to the principal investigator sylwester.kujach@gumed.edu.pl on reasonable request.

## References

[1] Degoutte F (2003). Energy demands during a judo match and recovery. Br. J. Sports Med..

[2] Franchini E, Del Vecchio FB, Matsushigue KA, Artioli GG (2011). Physiological profiles of elite judo athletes. Sports Med.

[3] Callister R (1991). Physiological characteristics of elite judo athletes. Int J Sports Med.

[4] Rossi C (2022). The role of psychological factors in judo: a systematic review. Int. J. Environ. Res. Public. Health.

[5] Wolska-Paczoska B (2010). The level of aerobic and anaerobic capacity and the results of a special mobility fitness test of female judo competitors aged 16–18 years. Balt. J. Health Phys. Act..

[6] Berkovich B-E, Eliakim A, Nemet D, Stark AH, Sinai T (2016). Rapid weight loss among adolescents participating in competitive judo. Int. J. Sport Nutr. Exerc. Metab..

[7] Burke, L. M. & Cox, G. R. Nutrition in Combat Sports. in *Combat Sports Medicine* (eds. Kordi, R., Maffulli, N., Wroble, R. R. & Wallace, W. A.) 1–20 (Springer, 2009). 10.1007/978-1-84800-354-5_1.

[8] Langan-Evans C, Close GL, Morton JP (2011). Making weight in combat sports. Strength Cond. J..

[9] Matthews JJ, Stanhope EN, Godwin MS, Holmes MEJ, Artioli GG (2019). The magnitude of rapid weight loss and rapid weight gain in combat sport athletes preparing for competition: a systematic review. Int. J. Sport Nutr. Exerc. Metab..

[10] Artioli GG (2010). Rapid weight loss followed by recovery time does not affect judo-related performance. J. Sports Sci..

[11] Giannini Artioli G (2010). Prevalence, magnitude, and methods of rapid weight loss among judo competitors. Med. Sci. Sports Exerc..

[12] Brito CJ (2012). Methods of body-mass reduction by combat sport athletes. Int. J. Sport Nutr. Exerc. Metab..

[13] Degoutte F (2006). Food restriction, performance, biochemical, psychological, and endocrine changes in judo athletes. Int. J. Sports Med..

[14] Kons, R. L., Da Silva Athayde, M. S., Follmer, B. & Detanico, D. Methods and magnitudes of rapid weight loss in judo athletes over pre-competition periods. *Hum. Mov.* **18**, (2017).

[15] Štangar M, Štangar A, Shtyrba V, Cigić B, Benedik E (2022). Rapid weight loss among elite-level judo athletes: Methods and nutrition in relation to competition performance. J. Int. Soc. Sports Nutr..

[16] Baribeau V (2023). Rapid weight gain and weight differential predict competitive success in 2100 professional combat-sport athletes. Int. J. Sports Physiol. Perform..

[17] Cannataro R, Cione E, Gallelli L, Marzullo N, Bonilla DA (2020). Acute effects of supervised making weight on health markers, hormones and body composition in Muay Thai fighters. Sports.

[18] Artioli GG (2010). The need of a weight management control program in judo: a proposal based on the successful case of wrestling. J. Int. Soc. Sports Nutr..

[19] Hyperthermia and Dehydration-Related Deaths Associated With Intentional Rapid Weight Loss in Three Collegiate Wrestlers—North Carolina, Wisconsin, and Michigan, November-December 1997. *JAMA* **279**, 824 (1998). 9480411

[20] Oppliger RA, Utter AC, Scott JR, Dick RW, Klossner D (2006). NCAA rule change improves weight loss among National Championship Wrestlers. Med. Sci. Sports Exerc..

[21] Calmet M, Pierantozzi E, Sterkowicz S, Takito MY, Franchini E (2017). Judo rules: searching for a wind of changes. Int. J. Perform. Anal. Sport.

[22] Ceylan B, Barley OR, Balci SS (2023). Changes in body mass and hydration status in judo athletes before and after a top-level competition: a descriptive case study. Phys. Sportsmed..

[23] Pettersson S, Ekström MP, Berg CM (2013). Practices of weight regulation among elite athletes in combat sports: A matter of mental advantage?. J. Athl. Train..

[24] Artioli GG, Saunders B, Iglesias RT, Franchini E (2016). It is time to ban rapid weight loss from combat sports. Sports Med..

[25] Malliaropoulos N (2019). Prevalence, techniques and knowledge of rapid weight loss amongst adult british judo athletes: A questionnaire based study. Muscle Ligaments Tendons J..

[26] Lakicevic N (2020). Effects of rapid weight loss on judo athletes: A systematic review. Nutrients.

[27] Roklicer R (2020). The effects of rapid weight loss on skeletal muscle in judo athletes. J. Transl. Med..

[28] Berkovich B-E, Stark AH, Eliakim A, Nemet D, Sinai T (2019). Rapid weight loss in competitive judo and taekwondo athletes: Attitudes and practices of coaches and trainers. Int. J. Sport Nutr. Exerc. Metab..

[29] Alderman BL, Landers DM, Carlson J, Scott JR (2004). Factors related to rapid weight loss practices among international-style wrestlers. Med. Sci. Sports Exerc..

[30] Selected Issues for Nutrition and the Athlete (2013). A team physician consensus statement. Med. Sci. Sports Exerc..

[31] Reale R, Cox GR, Slater G, Burke LM (2016). Regain in body mass after weigh-in is linked to success in real life judo competition. Int. J. Sport Nutr. Exerc. Metab..

[32] Reale R, Slater G, Burke LM (2017). Acute-weight-loss strategies for combat sports and applications to olympic success. Int. J. Sports Physiol. Perform..

[33] Meignié A (2021). The effects of menstrual cycle phase on elite athlete performance: A critical and systematic review. Front. Physiol..

[34] Armstrong LE (2007). Assessing hydration status: The elusive gold standard. J. Am. Coll. Nutr..

[35] Popowski LA (2001). Blood and urinary measures of hydration status during progressive acute dehydration. Med. Sci. Sports Exerc..

[36] Zambraski EJ, Tipton CM, Jordon HR, Palmer WK, Tcheng TK (1974). Iowa wrestling study: Urinary profiles of state finalists prior to competition. Med. Sci. Sports.

[37] Hamouti N, Del Coso J, Mora-Rodriguez R (2013). Comparison between blood and urinary fluid balance indices during dehydrating exercise and the subsequent hypohydration when fluid is not restored. Eur. J. Appl. Physiol..

[38] Fernández-Elías VE (2014). Validity of hydration non-invasive indices during the weightcutting and official weigh-in for Olympic combat sports. PLoS ONE.

[39] Shirreffs SM (2003). Markers of hydration status. Eur. J. Clin. Nutr..

[40] Armstrong LE (1994). Urinary indices of hydration status. Int. J. Sport Nutr..

[41] Exercise and Fluid Replacement (2007). Med. Sci. Sports Exerc..

[42] Olek RA, Kujach S, Wnuk D, Laskowski R (2014). Single sodium pyruvate ingestion modifies blood acid-base status and post-exercise lactate concentration in humans. Nutrients.

