# Supplementary material for: Examining the effects of pre-competition rapid weight loss on hydration status and competition performance in elite judo athletes
Source: Sci Rep. 2023 Sep 7;13:14756. doi: 10.1038/s41598-023-41872-1 (PMC10484915; doi:10.1038/s41598-023-41872-1)
Supplement: Supplementary file 1 — Supplementary Tables. [file 41598_2023_41872_MOESM1_ESM.pdf]

Supplementary material:

Examining the effects of pre-competition rapid weight loss on hydration status and competition performance in elite judo athletes

Table 1. Participants characteristics.

**Variable**	**Judo (n=17)**
Age [y]	18.7 ± 0.5
Height [cm]	172.2 ± 4.6
Weight [kg]	67.7 ± 5.2
Competition weight category [kg]	55* – 73
Judo experience (degree)	1 Kyu – 1 Dan

Values are mean ± SD., * Two individuals additionally participate in the youth category (under 20), approved by the Polish Judo Association (~55kg). Furthermore, youth participants also engage in seniors competition.

Table 2. The effect of RWL on body mass changes [kg], and body mass changes relative to the upper limit of the judo athlete's (n=17) weight category [%] within the five days prior to the competition.

Variables	-5 day	-1 day	Weight-in	Competition
Body mass [kg]	67.7 ± 5.2	65.1 ± 4.7	64.5 ± 4.7	67.0 ± 4.9*
Weight category [%]	105.1 ± 1.9	101.1 ± 1.1	100.1 ± 1.0	103.9 ± 1.5*

Body mass changes [kg], and body mass changes relative to the upper limit of the athlete's weight category [%] within the five days prior to the competition. Values are mean ± SD. * significant differences between groups (One way Anova with RM $p<0.0001$)

Bonferroni's post-hoc test

**Body mass**	Mean Diff.	t	p<0.05?	Summary	95% CI of diff
-5 day vs -1 day	2,565	9,472	Yes	***	1.820 to 3.310
-5 day vs Weight In	3,244	11,98	Yes	***	2.499 to 3.989
-5 day vs Competition	0,7647	2,824	Yes	*	0.01953 to 1.510
-1 day vs Weight In	0,6794	2,509	No	ns	-0.06577 to 1.425
-1 day vs Competition	-1,800	6,648	Yes	***	-2.545 to -1.055
Weight In vs Competition	-2,479	9,157	Yes	***	-3.225 to -1.734

Bonferroni's post hoc test

**Weight category**	Mean Diff.	t	p<0.05?	Summary	95% CI of diff
-5 day vs -1 day	3,995	9,856	Yes	***	2.879 to 5.110
-5 day vs Weight In	5,014	12,37	Yes	***	3.898 to 6.129
-5 day vs Competition	1,169	2,884	Yes	*	0.05342 to 2.284
-1 day vs Weight In	1,019	2,514	No	ns	-0.09658 to 2.134
-1 day vs Competition	-2,826	6,972	Yes	***	-3.941 to -1.710
Weight In vs Competition	-3,845	9,486	Yes	***	-4.960 to -2.729

Table 3. The effect of RWL on judo athletes (n=12) hydration status.

Variables	Before weight-in	Competition	After warm-up
U_{OSM} [mOsmol*kg]$^{-1}$	1005 ± 215.2	960.8 ± 243.7	884.8 ± 273.4ns
U_{sg} [g*cm3]$^{-1}$	1.031 ± 0.006	1.030 ± 0.007	1.027 ± 0.008ns

U_{OSM}- Urine osmolality [mOsmol*kg]$^{-1}$, and U_{sg}- Urine specific gravity [g*cm^3]$^{-1}$ changes before weigh-in, competition day, and after warm-up. Values are mean ± SD. ns- non-significant

Bonferroni's post-hoc test

**Urine osmolality**	Mean Diff.	t	p<0.05?	Summary	95% CI of diff
Before weight-in vs Competition	43,75	0,8050	No	ns	-97.08 to 184.6
Before weight-in vs After warm-up	119,7	2,202	No	ns	-21.16 to 260.5
Competition vs After warm-up	75,92	1,397	No	ns	-64.91 to 216.7

Bonferroni's post-hoc test

**Urine specific gravity**	Mean Diff.	t	p<0.05?	Summary	95% CI of diff
Before weight-in vs Competition	0,001000	0,5740	No	ns	-0.003514 to 0.005515
Before weight-in vs After warm-up	0,003500	2,009	No	ns	-0.001015 to 0.008014
Competition vs After warm-up	0,002500	1,435	No	ns	-0.002015 to 0.007014

Table 4. Rapid weight loss (RWL) methods among participants (%)

Variables	Prevalence [%]
Reduce total food volume	100
Layers of clothing	64
Low carbohydrate diet	53
Sauna/ Hot water bath	29
Water loading	24
Laxative	12
Herbal diuretics	12
Pharmacological diuretics	6
